# Annotations

**Published:** 1891-09-12

**Authors:** 


### Annotations.

**The Workman and the Hospital.**

That Hospitals are waking up to the necessity of dispensing their charities with something like common sense and justice is shown by the following instructive case which has gone the round of the newspapers. We give first the facts as they were represented in an evening paper, and secondly as they actually happened. According to a correspondent of the paper in question, "a workman employed in a cabinet manufactory in Holloway got a severe blow on one of his shins from a block of wood which flew out of the machine at which he was working. It inflicted a nasty bruise in addition to a not inconsiderable wound, which bled pretty freely. After washing and bandaging it he went to the Great Northern Central Hospital, arriving there about twelve o'clock. He was told to come in at one o'clock. When he went again at one o'clock he was interrogated by the enquiry officer as to age, residence, wages earned, whether in constant work, etc. After jotting down all the replies, the enquiry officer coolly informed him that his was a case for a private doctor, as he was in receipt of more than 30s. per week. The injured man explained that he came to the hospital believing that he was likely to get better treatment there than elsewhere, because the hospital doctors had daily experience in the treatment of such cases. He added that he and his fellow workmen always subscribed to the hospital funds, but the official had but one reply, 'Yours is a case for a private doctor.' Consequently the injured man had to leave the hospital after being kept hanging about for nearly two hours, and to see a private doctor. How serious or how trivial the injury was the hospital people apparently neither knew nor cared. None of them took the trouble to examine the injured limb." There are three points to be noted in this statement: first, that the injured man went to the hospital immediately after the accident, only waiting to wash and bandage the wound; secondly, that he was seen by the enquiry officer only, and dismissed on the ground of unfitness because his wages were thirty shillings a week; thirdly, that he was not examined by one of the hospital doctors at all, and that the hospital officials sent him away neither knowing nor caring what the nature of his injury might be. Such are the statements of the newspaper correspondent. We will now compare them with the actual facts. In the first place the applicant was seen and examined by Mr. W. A. Malcolm, M.B., the honorary medical officer specially appointed by the hospital to examine and report upon all such cases; secondly, the injury was of three or four days' standing, instead of being an accident case which had happened within an hour or two; thirdly, the man told Mr. Malcolm that he never earned less than thirty-five shillings a-week; that he was a cabinetmaker at Maple's factory, in constant work; that he was a single man, and had nobody dependent on him. Fourthly, that as he subscribed in his workshop to the Hospital Saturday Fund, he considered he was entitled to treatment. This is a kind of circumstance which is constantly happening, and we shall venture to express our opinion with all possible plainness. In our judgment a workman in Wilkinson's position, with constant work, excellent wages, and no dependants, ought to be ashamed of himself for seeking charitable medical relief under such trivial circumstances. He is evidently either a stupid or a shameless creature who has no English pride. He reminds us of the two impudent blockheads who broke Mr. Benson's windows the other day on the ground that they were in pecuniary distress, although sixteen shillings were found in their pockets. Such a man as Wilkinson had no more claim to hospital treatment under his then circumstances than the Duke of Westminster. And if he had been a man of any self-respect, he would no more have asked for charitable relief than the Duke would. The Great Northern Central Hospital has rendered a service to the public by its refusal to give Wilkinson charitable relief. That hospital has adopted an excellent system of out-patient administration. It employs not only the usual inquiry officer, but also a competent medical man to report upon all out-patient casualties. By this system no case requiring urgent aid is overlooked, and selfish fellows of the Wilkinson type are sent about their business like the sturdy beggars whose ranks they are so well fitted to join. The plea of obtaining better treatment at a hospital than an ordinary doctor could give for a bruised shin is too transparently absurd. If there be any doctor in London, or the country, who does not know how to treat a bruised shin, he ought to be put into a glass case and exhibited as a pathological specimen at the College of Surgeons Museum.

**Lecturers, Note-takers, and Books.**

The average medical lecturer expects his students to take copious notes—to take down, indeed, his lectures verbatim. The average student, to please his professor, does what is wished. Of all the methods of wasting time, and frustrating the true ends of lecturing, this of verbatim note-taking is the most successful. If a given professor is so impressed with the superiority of his own lectures over those of every other professor—and there are many lecturers who have reached even this sublime elevation of stupidity—is not the printing-press open to him? And can any student take down spoken lectures with half the fulness and accuracy with which they can be furnished by the printer? What is the object of lecturing? Is it not that the speaker may do what the written book cannot do—viz., supply the indispensable personal equation? The insistence upon the taking of verbatim notes is simply the converting of each student into an inaccurate, incomplete, and misleading printing-press. The lecturer is not a book—he never can be a book. He is a teacher. He supplies emphasis, measure, enthusiasm, the influence of eye and voice and hand. He is invaluable if he be a born teacher. If he be a mere repeater, even of lectures of his own composing, he is no better than a maddening parrot. The writer knows lecturers who are scientific and accurate enough, but who cannot teach. They simply meander for an hour through a mass of more or less relevant matter of a medical or scientific kind. What a miserable daily hour it is which is spent in the class-room of such a man! How one would like to take him into a grocer's shop, and tell him to wrap up pounds of sugar, or to cast up the price of a barrel of soap, making due allowance for tare! There is no subject which demands born teachers so much as medicine. The ground which has to be covered in five years is enormous and appalling. Medical education, in the true sense of the word, is almost unknown in many medical schools; medical cram of the most stupid and miserable kind is almost universal. Here and there a medical lecturer has so far conquered his own egotism as to suggest to his hearers the use of a text-book, sometimes not of his own writing. He will follow, more or less, the order of the text-book in his lectures. At the close of each day's lecture he will tell his class what he intends to lecture upon next day. All the intelligent members of the class will carefully read up the subject of the day's lecture beforehand; then, when the lecture hour comes round, they will stuff their note-books carefully into their pockets and give their undivided and critical attention to the speaker. If the professor have anything new to say, he will inform his hearers of the fact, and they will take a carefully written note of it in their note-books. Such lecturers as these know how to teach. Half a dozen lectures by one of them do more for the real education and training of the medical student than half a hundred where verbatim notes are taken. If medical lecturers are so unintelligent and out of date as to insist upon verbatim notes, the students, in a body, should strike against such vain, childish, and profitless methods of work. There is much need of a revolution in almost all our medical schools.

